# Supplementary material for: Histologic and Immunohistochemical Patterns in Lymphomatoid Papulosis: A Systematic Review of Published Cases
Source: Dermatopathology (Basel). 2025 Feb 12;12(1):6. doi: 10.3390/dermatopathology12010006 (PMC11861998; doi:10.3390/dermatopathology12010006)
Supplement: Supplementary file 1 [file dermatopathology-12-00006-s001.zip › dermatopathology-3429193-supplementary.pdf]

Supplemental data

Suppl. Table S1: All investigated histologic patterns, immunophenotypic and genetic features, which could be extracted from 13 articles with diagnosed Lyp subtype A cases. The highlighted lines show the key-features we extracted showed in the figures.

	Willemze (1982)* (55)	Barnadas (1992) (43)	El Shabrawi-Caelen (2004)*(20)	Flann (2006) (51)	Sharma (2007) (48)	de Souza (2009) (47)	de Souza (2012) (21)	Dumke (2013) (49)	Booken (2013) (53)	Martinez-Escala (2014) (54)	Marschalko (2016) (23)	Sanchez-España (2019) (52)	Kavvalou (2019) (50)	Total
**number of examined specimen**	45	1	104	2	1	12	93	1	1	2	1	1	1	265
**number of patients**	13	1	64	2	1	12	69	1	1	2	1	1	1	169
**age of patients (years)**														
mean	43,2	16	47	35	21	12	45	60	85	64	33	37	27	40,40
min	24	16	4	23	21	19	1	60	85	57	33	37	27	1,00
max	71	16	84	47	21	1	83	60	85	71	33	37	27	85
**sex of patients**														
male	8	0	41	1	1	7	?	1	1	2	1	1	1	65
female	5	1	23	1	0	5	?	0	0	0	0	0	0	35
divers	0	0	0	0	0	0	?	0	0	0	0	0	0	0
**histological features**														
epidermotropic (basal)	+/++	1 (100%)	55 (53%)	N/A	N/A	7 (58%)	54 (58%)	0 (0%)	0 (0%)	0 (0%)	1 (100%)	0 (0%)	0 (0%)	118/217 (54%)
epidermotropic (pagetoid)	-	0 (0%)	0 (0%)	N/A	1 (100%)	0 (0%)	2 (2%)	0 (0%)	0 (0%)	0 (0%)	N/A	0 (0%)	0 (0%)	3/217 (1%)
dermal	++	1 (100%)	104 (100%)	2 (100%)	1 (100%)	12 (100%)	93 (100%)	1 (100%)	1 (100%)	2 (100%)	1 (100%)	1 (100%)	1 (100%)	220/220 (100%)
perifollicular	N/A	N/A	5 (5%)	N/A	N/A	N/A	46 (49%)	1 (100%)	N/A	N/A	1 (100%)	N/A	N/A	53/199 (27%)
folliculotropic	N/A	N/A	5 (5%)	N/A	N/A	N/A	46 (49%)	N/A	N/A	N/A	N/A	N/A	N/A	51/197 (26%)
angiocentric	-	N/A	+	N/A	N/A	N/A	N/A	N/A	N/A	1 (50%)	N/A	N/A	N/A	1/2 (50%)
perivascular	+	1 (100%)	+	1 (50%)	N/A	4 (33%)	N/A	1 (100%)	N/A	1 (50%)	1 (100%)	1 (100%)	N/A	10/20 (50%)
vascular occlusion	N/A	N/A	N/A	N/A	N/A	4 (33%)	24 (26%)	N/A	N/A	0 (0%)	N/A	N/A	N/A	28/107 (26%)
hemorrhage	-	N/A	N/A	N/A	N/A	7 (58%)	50 (54%)	N/A	N/A	1 (50%)	N/A	N/A	N/A	58/107 (54%)
granulomatous	N/A	N/A	N/A	N/A	N/A	N/A	N/A	N/A	N/A	N/A	N/A	N/A	N/A	N/A
perisyringo	N/A	N/A	1 (1%)	N/A	N/A	7 (58%)	54 (58%)	1 (100%)	N/A	N/A	1 (100%)	N/A	N/A	64/211 (30%)
syringotropic	N/A	N/A	1 (1%)	N/A	N/A	9 (75%)	46 (49%)	N/A	N/A	N/A	N/A	N/A	N/A	56/209 (27%)
diffuse Infiltrate	+	1 (100%)	+	N/A	N/A	N/A	N/A	N/A	1 (100%)	N/A	N/A	N/A	1 (100%)	3/3 (100%)
single Infiltrate	N/A	N/A	N/A	N/A	N/A	N/A	N/A	N/A	0 (0%)	N/A	N/A	N/A	N/A	0/1 (0%)
cluster	N/A	N/A	N/A	N/A	N/A	N/A	N/A	N/A	N/A	N/A	N/A	N/A	N/A	N/A
nodular	N/A	N/A	N/A	N/A	N/A	N/A	N/A	N/A	1 (100%)	N/A	N/A	N/A	N/A	1/1 (100%)
cohesive sheets	N/A	N/A	N/A	N/A	N/A	N/A	N/A	N/A	N/A	N/A	N/A	N/A	N/A	N/A
small cells	+	N/A	15 (14%)	N/A	N/A	12 (100%)	N/A	0 (0%)	0 (0%)	0 (0%)	1 (100%)	N/A	1 (100%)	29/122 (24%)
medium cells	+	N/A	104 (100%)	N/A	N/A	0 (0%)	N/A	0 (0%)	1 (100%)	2 (100%)	0 (0%)	N/A	1 (100%)	108/122 (89%)
large cells	-	N/A	104 (100%)	2 (100%)	1 (100%)	0 (0%)	N/A	1 (100%)	1 (100%)	1 (50%)	1 (100%)	1 (100%)	1 (100%)	113/126 (90%)
monomorph cells	N/A	N/A	N/A	N/A	N/A	N/A	N/A	0 (0%)	N/A	N/A	N/A	N/A	N/A	0/1 (0%)
pleomorph cells	N/A	N/A	104 (100%)	N/A	N/A	N/A	N/A	1 (100%)	N/A	1 (50%)	N/A	N/A	N/A	106/107 (99%)
immunoblastic cells	N/A	N/A	N/A	N/A	N/A	N/A	N/A	N/A	N/A	N/A	N/A	N/A	N/A	N/A
anaplastic cells	N/A	N/A	N/A	1 (50%)	1 (100%)	12 (100%)	N/A	N/A	1 (100%)	1 (50%)	N/A	N/A	1 (100%)	17/19 (90%)
multinucleated cells (Reed-Sternberg-like)	++	1 (100%)	N/A	N/A	1 (100%)	N/A	N/A	1 (100%)	N/A	1 (50%)	N/A	N/A		4/5 (80%)
eosinophils	++	1 (100%)	70 (67%)	N/A	N/A	7 (58%)	50 (54%)	1 (100%)	1 (100%)	1 (50%)	0 (0%)	1 (100%)	0 (0%)	132/217 (61%)
neutrophils	++	1 (100%)	86 (83%)	N/A	N/A	7 (58%)	33 (35%)	0 (0%)	0 (0%)	0 (0%)	0 (0%)	N/A	1 (100%)	128/216 (60%)
histiocytes	++	N/A	N/A	N/A	N/A	N/A	N/A	0 (0%)	1 (100%)	0 (0%)	N/A	1 (100%)	1 (100%)	3/6 (50%)
small lymphocytes (reactive)	+	1 (100%)	N/A	N/A	N/A	12 (100%)	93 (100%)	1 (100%)	1 (100%)	0 (0%)	1 (100%)	1 (100%)	1 (100%)	111/113 (98%)
plasma cells	N/A	N/A	N/A	N/A	N/A	N/A	N/A	0 (0%)	N/A	N/A	N/A	N/A	N/A	0/1 (0%)
**immunophenotype**														
ALK	N/A	N/A	N/A	N/A	N/A	N/A	N/A	N/A	N/A	N/A	N/A	0 (0%)	0 (0%)	0/2 (0%)
CD2+	N/A	N/A	N/A	2 (100%)	N/A	N/A	N/A	N/A	N/A	N/A	N/A	N/A	N/A	2/2 (100%)
CD3+	N/A	N/A	N/A	2 (100%)	N/A	12 (100%)	N/A	1 (100%)	1 (100%)	N/A	N/A	1 (100%)	1 (100%)	18/18 (100%)
CD5+	N/A	N/A	N/A	N/A	N/A	N/A	N/A	N/A	N/A	0/1 (0%)	N/A	1 (100%)	N/A	1/3 (33%)
**CD4+**/CD8-	N/A	N/A	5/16 (31,2%)	0 (0%)	N/A	1 (8,3%)	45 (48,4%)	0 (0%)	1 (100%)	0 (0%)	0 (0%)	1 (100%)	1 (100%)	54/130 (42%)
CD4-/**CD8+**	N/A	N/A	6/16 (37,5%)	2 (100%)	N/A	10 (83,3%)	48 (51,6%)	0 (0%)	0 (0%)	2 (100%)	1 (100%)	0 (0%)	0 (0%)	69/130 (53%)
**CD4+/CD8+**	N/A	N/A	1/16 (6,3%)	0 (0%)	N/A	0 (0%)	0 (0%)	1 (100%)	0 (0%)	0 (0%)	0 (0%)	0 (0%)	0 (0%)	2/130 (2%)
CD4-/CD8-	N/A	N/A	4/16 (25%)	0 (0%)	N/A	1 (8,3%)	0 (0%)	0 (0%)	0 (0%)	0 (0%)	0 (0%)	0 (0%)	0 (0%)	5/130 (4%)
CD15+	N/A	N/A	N/A	N/A	N/A	N/A	N/A	N/A	N/A	N/A	N/A	N/A	0 (0%)	0/1 (0%)
CD20+	N/A	N/A	N/A	N/A	N/A	N/A	N/A	N/A	N/A	N/A	N/A	N/A	N/A	N/A
CD30+	N/A	N/A	68/68 (100%)	2 (100%)	1 (100%)	12 (100%)	93 (100%)	1 (100%)	1 (100%)	1/1 (100%)	1 (100%)	1 (100%)	1 (100%)	182/182 (100%)
CD45+	N/A	N/A	N/A	N/A	N/A	N/A	N/A	N/A	N/A	0/1 (0%)	N/A	N/A	N/A	0/2 (0%)
CD56+	N/A	N/A	9/22 (41%)	2 (100%)	N/A	N/A	N/A	N/A	N/A	0/1 (0%)	N/A	N/A	N/A	11/26 (42%)
Ki-67	N/A	N/A	N/A	N/A	N/A	N/A	N/A	N/A	N/A	N/A	N/A	50%	80%	50-80%
TCR-alpha/beta	N/A	N/A	N/A	N/A	N/A	N/A	N/A	N/A	N/A	0/1 (0%)	N/A	N/A	N/A	0/1 (0%)
TCR-gamma/delta	N/A	N/A	N/A	N/A	N/A	N/A	N/A	N/A	N/A	1/1 (100%)	N/A	N/A	N/A	1/1 (100%)
TIA-1/granzyme B/perforin	N/A	N/A	15/21 (71%)	2 (100%)	N/A	3 (25%)	N/A	N/A	N/A	1/1 (100%)	N/A	N/A	1 (100%)	22/37 (59%)
DUSP22-IRF4	N/A	N/A	N/A	N/A	N/A	N/A	N/A	N/A	N/A	N/A	N/A	N/A	N/A	N/A

Suppl. Table S2: All investigated histologic patterns, immunophenotypic and genetic features, which could be extracted from 11 articles with diagnosed Lyp subtype B cases. The highlighted lines show the key-features we extracted showed in the figures.

	Willemze (1982) (55)	ElShabrawi-Caelen (2004) (20)	Flann (2006) (51)	de Souza (2009) (47)	Criscuoli (2012) (56)	de Souza (2012) (21)	Plaza (2013) (22)	Martinez-Escala (2014) (54)	Marshalko (2016) (23)	Bouhamidi (2018) (58)	Medvez (2019) (57)	Total
**number of examined specimen**	19	8	1	2	1	31	3	2	4	1	1	73
**number of patients**	7	3	1	1	1	13	3	2	4	1	1	37
**age of patients (years)**												
mean	33,9	35	29	17	38	62	54,3	41,5	38,5	28	38	37,75
min	24	29	29	17	38	21	34	30	6	28	38	6
max	64	54	29	17	38	83	77	53	69	28	38	83
**sex of patients**												
male	5	3	1	1	1	?	2	0	4	1	1	19
female	2	0	0	0	0	?	1	2	0	0	0	5
divers	0	0	0	0	0	?	0	0	0	0	0	0
**histological features**												
epidermotropic (basal)	++	8 (100%)	1 (100%)	2 (100%)	N/A	31 (100%)	3 (100%)	2 (100%)	4 (100%)	N/A	1 (100%)	53/53 (100%)
epidermotropic (pagetoid)	N/A	N/A	N/A	N/A	N/A	20 (65%)	N/A	0 (0%)	0 (0%)	N/A	0 (0%)	20/38 (53%)
dermal	++	8 (100%)	1 (100%)	2 (100%)	1 (100%)	31 (100%)	3 (100%)	2 (100%)	4 (100%)	N/A	1 (100%)	53/53 (100%)
perifollicular	N/A	N/A	N/A	1 (50%)	N/A	14 (47%)	N/A	N/A	1 (25%)	N/A	1 (100%)	17/38 (45%)
folliculotropic	N/A	N/A	N/A	1 (50%)	N/A	14 (47%)	N/A	N/A	N/A	N/A	N/A	15/33 (46%)
angiocentric	N/A	N/A	N/A	N/A	N/A	N/A	2 (66,7%)	N/A	N/A	N/A	N/A	2/3 (67%)
perivascular	+	N/A	N/A	N/A	N/A	N/A	N/A	N/A	4 (100%)	N/A	1 (100%)	5/5 (100%)
vascular occlusion	N/A	N/A	N/A	0 (0%)	N/A	8 (26%)	N/A	N/A	N/A	N/A	N/A	8/33 (24%)
hemorrhage	-	N/A	N/A	0 (0%)	N/A	12 (39%)	N/A	0 (0%)	N/A	N/A	1 (100%)	13/36 (36%)
granulomatous	N/A	N/A	N/A	N/A	N/A	N/A	N/A	N/A	N/A	N/A	N/A	N/A
perisyringo	N/A	N/A	N/A	1 (50%)	N/A	13 (42%)	0 (0%)	N/A	1 (25%)	N/A	1 (100%)	16/41 (39%)
syringotropic	N/A	N/A	N/A	1 (50%)	N/A	13 (42%)	0 (0%)	N/A	N/A	N/A	N/A	14/36 (39%)
diffuse Infiltrate	-	N/A	N/A	N/A	1 (100%)	N/A	N/A	2 (100%)	N/A	N/A	N/A	3/3 (100%)
single Infiltrate	N/A	N/A	N/A	N/A	N/A	N/A	N/A	0 (0%)	N/A	N/A	N/A	0/2 (0%)
cluster	N/A	N/A	N/A	N/A	N/A	N/A	N/A	N/A	N/A	N/A	N/A	N/A
nodular	N/A	N/A	N/A	N/A	N/A	N/A	N/A	N/A	N/A	N/A	N/A	N/A
cohesive sheets	N/A	N/A	N/A	N/A	N/A	N/A	N/A	N/A	N/A	N/A	N/A	N/A
small cells	-	8 (100%)	0 (0%)	1 (50%)	0 (0%)	N/A	0 (0%)	1 (50%)	4 (100%)	N/A	1 (100%)	15/22 (68%)
medium cells	-	8 (100%)	1 (100%)	0 (0%)	0 (0%)	N/A	0 (0%)	1 (50%)	0 (0%)	N/A	1 (100%)	11/22 (50%)
large cells	+	N/A	0 (0%)	0 (0%)	1 (100%)	7 (23%)	1 (33,3%)	2 (100%)	0 (0%)	1 (100%)	0 (0%)	12/46 (26%)
monomorph cells	N/A	N/A	N/A	N/A	N/A	N/A	1 (33,3%)	2 (100%)	N/A	N/A	N/A	3/5 (60%)
pleomorph cells	N/A	N/A	N/A	N/A	N/A	N/A	0 (0%)	0 (0%)	N/A	N/A	N/A	0/5 (0%)
immunoblastic cells	N/A	N/A	N/A	N/A	N/A	N/A	1 (33,3%)	0 (0%)	N/A	N/A	N/A	1/5 (20%)
anaplastic cells	N/A	N/A	N/A	N/A	N/A	N/A	N/A	2 (100%)	N/A	N/A	N/A	2/2 (100%)
multinucleated cells (Reed-Sternberg-like)	N/A	N/A	N/A	N/A	N/A	N/A	N/A	0 (0%)	N/A	N/A	N/A	0/2 (0%)
eosinophils	+	3 (38%)	N/A	1 (50%)	1 (100%)	8 (26%)	2 (66,7%)	1 (50%)	0 (0%)	N/A	N/A	16/50 (32%)
neutrophils	+	5 (63%)	N/A	0 (0%)	0 (0%)	8 (26%)	1 (33,3%)	0 (0%)	0 (0%)	N/A	N/A	14/50 (30%)
histiocytes	+	N/A	N/A	N/A	1 (100%)	N/A	N/A	0 (0%)	N/A	N/A	N/A	1/3 (33%)
small lymphocytes (reactive)	++	N/A	N/A	N/A	1 (100%)	N/A	1 (33,3%)	1 (50%)	4 (100%)	N/A	N/A	7/9 (78%)
plasma cells	-	N/A	N/A	N/A	0 (0%)	N/A	N/A	N/A	N/A	N/A	N/A	0/1 (0%)
**immunophenotype**												
ALK	N/A	N/A	N/A	N/A	N/A	N/A	0 (0%)	N/A	N/A	N/A	N/A	0/3 (0%)
CD2+	N/A	N/A	1 (100%)	N/A	N/A	N/A	3 (100%)	N/A	4 (100%)	N/A	N/A	8/8 (100%)
CD3+	N/A	N/A	1 (100%)	N/A	N/A	N/A	3 (100%)	1/1 (100%)	N/A	N/A	1 (100%)	6/6 (100%)
CD5+	N/A	N/A	N/A	N/A	N/A	N/A	1/2 (50%)	0 (0%)	4 (100%)	N/A	N/A	5/8 (63%%)
**CD4+**/CD8-	N/A	1/2 (50%)	0 (0%)	1 (50%)	N/A	17 (54,8%)	0 (0%)	0 (0%)	0 (0%)	N/A	1 (100%)	20/46 (43%)
CD4-/**CD8+**	N/A	0/2 (0%)	1 (100%)	1 (50%)	N/A	14 (45,2%)	3 (100%)	1 (50%)	4 (100%)	N/A	0 (0%)	24/46 (52%)
**CD4+/CD8+**	N/A	1/2 (50%)	0 (0%)	0 (0%)	N/A	0 (0%)	0 (0%)	0 (0%)	0 (0%)	N/A	0 (0%)	1/46 (2%)
CD4-/CD8-	N/A	0/2 (0%)	0 (0%)	0 (0%)	N/A	0 (0%)	0 (0%)	1 (50%)	0 (0%)	N/A	0 (0%)	1/46 (2%)
CD15+	N/A	N/A	N/A	N/A	N/A	N/A	N/A	N/A	N/A	N/A	N/A	N/A
CD20+	N/A	N/A	N/A	N/A	N/A	N/A	N/A	N/A	N/A	N/A	N/A	N/A
CD30+	N/A	3/5 (60%)	0 (0%)	0 (0%)	1 (100%)	31 (100%)	3 (100%)	2 (100%)	2 (50%)	1 (100%)	1 (100%)	44/51 (86%)
CD45+	N/A	N/A	N/A	N/A	N/A	N/A	N/A	N/A	N/A	N/A	N/A	N/A
CD56+	N/A	1/2 (50%)	1 (100%)	N/A	N/A	N/A	0 (0%)	2 (100%)	N/A	N/A	N/A	4/8 (50%)
Ki-67	N/A	N/A	N/A	N/A	N/A	N/A	N/A	N/A	N/A	N/A	N/A	N/A
TCR-alpha/beta	N/A	N/A	N/A	N/A	N/A	N/A	N/A	N/A	N/A	N/A	N/A	N/A
TCR-gamma/delta	N/A	N/A	N/A	N/A	N/A	N/A	N/A	1/1 (100%)	N/A	N/A	N/A	1/1 (100%)
TIA-1/granzyme B/perforin	N/A	1/1 (100%)	1 (100%)	1 (50%)	N/A	N/A	1/2 (50%)	2 (100%)	3 (75%)	N/A	N/A	9/12 (75%)
DUSP22-IRF4	N/A	N/A	N/A	N/A	N/A	N/A	N/A	N/A	N/A	N/A	N/A	N/A

Suppl. Table S3: All investigated histologic patterns, immunophenotypic and genetic features, which could be extracted from 10 articles with diagnosed Lyp subtype C cases. The highlighted lines show the key-features we extracted showed in the figures.

	Plettenberg (2003) (59)	El Shabrawi-Caelen (2004) (20)	de Souza (2012) (21)	Plaza (2013) (22)	Martinez-Escala (2014) (54)	Meena (2014) (61)	Haus (2014) (60)	Marshalko (2016) (23)	Schwartz (2017) (62)	Wang (2021) (24)	Total
**number of examined specimen**	2	24	9	7	2	1	1	1	5	1	53
**number of patients**	2	11	6	7	2	1	1	1	5	1	37
**age of patients (years)**											
mean	36,5	38	49	57,6	46	14	22	24	52,6	N/A	37,74
min	10	7	17	17	44	14	22	24	34	N/A	7
max	63	68	70	91	48	14	22	24	71	N/A	91,00
**sex of patients**											
male	2	3	?	6	1	0	0	1	1	N/A	14
female	0	8	?	1	1	1	1	0	4	N/A	16
divers	0	0	?	0	0	0	0	0	0	N/A	0
**histological features**											
epidermotropic (basal)	1 (50%)	13 (54%)	6 (67%)	5 (71%)	0 (0%)	1 (100%)	1 (100%)	1 (100%)	2 (40%)	0 (0%)	30/53 (57%)
epidermotropic (pagetoid)	0 (0%)	0 (0%)	6 (67%)	1 (14%)	0 (0%)	0 (0%)	0 (0%)	0 (0%)	0 (0%)	0 (0%)	7/53 (13%)
dermal	2 (100%)	24 (100%)	9 (100%)	7 (100%)	2 (100%)	1 (100%)	1 (100%)	1 (100%)	2/2 (100%)	1 (100%)	51/51 (100%)
perifollicular	N/A	N/A	8 (89%)	N/A	N/A	N/A	N/A	0 (0%)	N/A	N/A	8/10 (80%)
folliculotropic	N/A	0 (0%)	8 (89%)	N/A	N/A	N/A	N/A	N/A	N/A	N/A	8/33 (24%)
angiocentric	N/A	N/A	N/A	4 (57%)	0 (0%)	N/A	N/A	1 (100%)	N/A	N/A	5/10 (50%)
perivascular	N/A	N/A	N/A	N/A	0 (0%)	1 (100%)	N/A	0 (0%)	2 (40%)	N/A	3/9 (33%)
vascular occlusion	N/A	N/A	5 (56%)	N/A	0 (0%)	N/A	N/A	N/A	N/A	N/A	5/11 (46%)
hemorrhage	N/A	N/A	8 (89%)	N/A	0 (0%)	N/A	N/A	N/A	N/A	N/A	8/11 (73%)
granulomatous	N/A	N/A	N/A	3 (43%)	0 (0%)	N/A	N/A	N/A	N/A	N/A	3/9 (33%)
perisyringo	N/A	N/A	7 (78%)	2 (29%)	N/A	N/A	N/A	0 (0%)	N/A	N/A	9/17 (53%)
syringotropic	N/A	N/A	N/A	N/A	N/A	N/A	N/A	N/A	N/A	N/A	N/A
diffuse Infiltrate	N/A	N/A	N/A	7 (100%)	N/A	1 (100%)	1 (100%)	N/A	N/A	N/A	9/9 (100%)
single Infiltrate	N/A	N/A	N/A	N/A	N/A	0 (0%)	0 (0%)	N/A	N/A	N/A	0/2 (0%)
cluster	N/A	N/A	N/A	N/A	N/A	N/A	1 (100%)	N/A	N/A	N/A	1/1 (100%)
nodular	N/A	N/A	N/A	7 (100%)	N/A	N/A	0 (0%)	1 (100%)	N/A	N/A	8/9 (89%)
cohesive sheets	N/A	N/A	N/A	N/A	N/A	N/A	1 (100%)	1 (100%)	1/1 (100%)	1 (100%)	4/4 (100%)
small cells	0 (0%)	5 (21%)	N/A	N/A	1 (50%)	0 (0%)	0 (0%)	0 (0%)	N/A	N/A	6/31 (19%)
medium cells	1 (50%)	24 (100%)	N/A	N/A	1 (50%)	1 (100%)	1 (100%)	0 (0%)	1 (20%)	N/A	29/36 (81%)
large cells	2 (100%)	24 (100%)	N/A	7 (100%)	1 (50%)	1 (100%)	1 (100%)	1 (100%)	5 (100%)	1 (100%)	43/44 (98%)
monomorph cells	N/A	N/A	N/A	7 (100%)	N/A	0 (0%)	1 (100%)	N/A	0 (0%)	N/A	8/14 (57%)
pleomorph cells	N/A	N/A	N/A	0 (0%)	2 (100%)	1 (100%)	1 (100%)	N/A	5 (100%)	1 (100%)	10/17 (59%)
immunoblastic cells	N/A	N/A	N/A	7 (100%)	N/A	N/A	N/A	N/A	N/A	N/A	7/7 (100%)
anaplastic cells	N/A	16 (66%)	N/A	N/A	2 (100%)	1 (100%)	N/A	1 (100%)	5 (100%)	1 (100%)	26/34 (76%)
multinucleated cells (Reed-Sternberg-like)	2 (100%)	14 (58%)	N/A	0 (0%)	N/A	1 (100%)	N/A	N/A	N/A	N/A	17/34 (50%)
eosinophils	2 (100%)	16 (66%)	5 (56%)	7 (100%)	0 (0%)	0 (0%)	N/A	0 (0%)	2 (40%)	1 (100%)	33/52 (64%)
neutrophils	2 (100%)	23 (95%)	4 (44%)	3 (43%)	0 (0%)	N/A	N/A	0 (0%)	1 (20%)	1 (100%)	34/51 (67%)
histiocytes	N/A	N/A	N/A	N/A	N/A	1 (100%)	N/A	0 (0%)	N/A	1 (100%)	2/3 (33%)
small lymphocytes (reactive)	N/A	N/A	9 (100%)	N/A	2 (100%)	1 (100%)	N/A	0 (0%)	1 (20%)	1 (100%	14/19 (74%)
plasma cells	N/A	N/A	N/A	N/A	N/A	0 (0%)	N/A	0 (0%)	N/A	0 (0%)	0/3 (0%)
**immunophenotype**											
ALK+	N/A	N/A	N/A	0 (0%)	N/A	0 (0%)	0 (0%)	N/A	N/A	N/A	0/9 (0%)
CD2+	N/A	N/A	N/A	3/3 (100%)	N/A	0 (0%)	N/A	N/A	N/A	1 (100%)	4/5 (80%)
CD3+	N/A	N/A	N/A	7 (100%)	1/1 (100%)	1 (100%)	0 (0%)	N/A	N/A	N/A	9/10 (90%)
CD5+	N/A	N/A	N/A	3/3 (100%)	0 (0%)	1 (100%)	N/A	N/A	0 (0%)	N/A	4/11 (36%)
**CD4+**/CD8-	N/A	1/3 (33,3%)	9 (100%)	0 (0%)	0 (0%)	N/A	0 (0%)	0 (0%)	4 (80%)	1 (100%)	15/29 (52%)
CD4-/**CD8+**	N/A	1/3 (33,3%)	0 (0%)	7 (100%)	2 (100%)	N/A	0 (0%)	1 (100%)	0 (0%)	0 (0%)	11/29 (38%)
**CD4+/CD8+**	N/A	1/3 (33,3%)	0 (0%)	0 (0%)	0 (0%)	N/A	0 (0%)	0 (0%)	0 (0%)	0 (0%)	1/29 (4%)
CD4-/CD8-	N/A	0/3 (0%)	0 (0%)	0 (0%)	0 (0%)	N/A	1 (100%)	0 (0%)	1 (20%)	0 (0%)	2/29 (7%)
CD15+	N/A	N/A	N/A	N/A	N/A	N/A	N/A	N/A	N/A	N/A	N/A
CD20+	N/A	N/A	N/A	N/A	N/A	0 (0%)	N/A	N/A	N/A	1 (100%)	1/2 (50%)
CD30+	2 (100%)	16/16 (100%)	9 (100%)	7 (100%)	2 (100%)	1 (100%)	1 (100%)	1 (100%)	5 (100%)	1 (100%)	53/53 (100%)
CD45+	N/A	N/A	N/A	N/A	1 (50%)	1 (100%)	N/A	N/A	N/A	1 (100%)	3/4 (75%)
CD56+	N/A	3/6 (50%)	N/A	2 (29%)	1 (50%)	N/A	N/A	N/A	N/A	N/A	6/15 (40%)
Ki-67	N/A	N/A	N/A	N/A	N/A	N/A	80%	N/A	N/A	90%	80-90%
TCR-alpha/beta	N/A	N/A	N/A	N/A	0/1 (0%)	N/A	N/A	N/A	N/A	N/A	0/1 (0%)
TCR-gamma/delta	N/A	N/A	N/A	N/A	2/2 (100%)	N/A	N/A	N/A	N/A	N/A	2/2 (100%)
TIA-1/granzyme B/perforin	N/A	4/5 (80%)	N/A	7 (100%)	1 (50%)	N/A	N/A	N/A	3 (60%)	N/A	15/19 (79%)
DUSP22-IRF4	N/A	N/A	N/A	N/A	N/A	N/A	N/A	N/A	N/A	N/A	N/A

Suppl. Table S4: All investigated histologic patterns, immunophenotypic and genetic features, which could be extracted from 19 articles with diagnosed Lyp subtype D cases. The highlighted lines show the key-features we extracted showed in the figures.

	Magro (2006) (64)	Saggini (2010) (25)	Cardoso (2012) (65)	Bertolotti (2013) (65)	Wen (2013) (26)	Plaza (2013) (22)	Martinez-Escala (2014) (54)	McQuitty (2014) (27)	Andersen (2014) (67)	Cho-Vega (2014) (68)
**number of examined specimen**	4	9	1	2	1	2	1	17	1	2
**number of patients**	4	9	1	2	1	2	1	17	1	2
**age of patients (years)**										
mean	35,75	27,2	38	56	23	53	57	52,6	48	?
min	14	10	38	50	23	41	57	11	48	?
max	61	38	38	62	23	65	57	91	48	?
**sex of patients**										
male	4	6	0	0	1	2	1	7	0	?
female	1	3	1	2	0	0	0	10	1	?
divers	0	0	0	0	0	0	0	0	0	?
**histological features**										
epidermotropic (basal)	4 (100%)	9 (100%)	1 (100%)	2 (100%)	1 (100%)	2 (100%)	N/A	4/11 (37%)	1 (100%)	2 (100%)
epidermotropic (pagetoid)	1/1 (100%)	9 (100%)	1 (100%)	2 (100%)	1 (100%)	2 (100%)	N/A	6/11 (55%)	1 (100%)	2 (100%)
dermal	4 (100%)	9 (100%)	1 (100%)	2 (100%)	1 (100%)	2 (100%)	1 (100%)	11/11 (100%)	1 (100%)	2 (100%)
perifollicular	3 (75%)	N/A	N/A	N/A	N/A	N/A	N/A	4/9 (44%)	N/A	N/A
folliculotropic	N/A	N/A	N/A	N/A	N/A	N/A	N/A	N/A	N/A	N/A
angiocentric	4 (100%)	N/A	N/A	N/A	N/A	0 (0%)	N/A	N/A	N/A	0 (0%)
perivascular	3 (75%)	9 (100%)	N/A	2 (100%)	1 (100%)	2 (100%)	N/A	11/11 (100%)	1 (100%)	0 (0%)
vascular occlusion	N/A	N/A	N/A	N/A	N/A	N/A	N/A	N/A	N/A	0 (0%)
hemorrhage	4 (100%)	N/A	N/A	N/A	N/A	N/A	1 (100%)	4/9 (44%)	N/A	0 (0%)
granulomatous	3 (75%)	N/A	N/A	N/A	N/A	0 (0%)	N/A	0 (0%)	N/A	N/A
perisyringo	3 (75%)	N/A	N/A	N/A	N/A	N/A	N/A	4/9 (44%)	N/A	N/A
syringotropic	N/A	N/A	N/A	N/A	N/A	N/A	N/A	N/A	N/A	N/A
diffuse Infiltrate	N/A	9 (100%)	1 (100%)	2 (100%)	1 (100%)	N/A	N/A	11/11 (100%)	1 (100%)	N/A
single Infiltrate	N/A	N/A	0 (0%)	2 (100%)	N/A	N/A	N/A	0 (0%)	0 (0%)	N/A
cluster	N/A	N/A	1 (100%)	2 (100%)	N/A	N/A	N/A	0 (0%)	0 (0%)	N/A
nodular	4 (100%)	0 (0%)	0 (0%)	0 (0%)	N/A	N/A	N/A	N/A	0 (0%)	N/A
cohesive sheets	N/A	N/A	N/A	N/A	N/A	N/A	N/A	2/10 (20%)	N/A	N/A
small cells	3 (75%)	0 (0%)	1 (100%)	N/A	N/A	2 (100%)	0 (0%)	8/10 (80%)	1 (100%)	0 (0%)
medium cells	1 (25%)	9 (100%)	1 (100%)	2 (100%)	1 (100%)	2 (100%)	1 (100%)	0/10 (0%)	0 (0%)	2 (100%)
large cells	1 (25%)	1 (11%)	1 (100%)	N/A	1 (100%)	0 (0%)	0 (0%)	8/10 (80%)	0 (0%)	2 (100%)
monomorph cells	4 (100%)	0 (0%)	0 (0%)	0 (0%)	N/A	2 (100%)	0 (0%)	0/10 (0%)	N/A	N/A
pleomorph cells	0 (0%)	9 (100%)	1 (100%)	2 (100%)	N/A	0 (0%)	1 (100%)	9/10 (90%)	1 (100%)	N/A
immunoblastic cells	N/A	0 (0%)	N/A	N/A	N/A	N/A	N/A	N/A	0 (0%)	N/A
anaplastic cells	0 (0%)	1 (11%)	N/A	N/A	N/A	N/A	N/A	N/A	0 (0%)	N/A
multinucleated cells (Reed-Sternberg-like)	0 (0%)	0 (0%)	1 (100%)	N/A	N/A	N/A	0 (0%)	N/A	N/A	N/A
eosinophils	4 (100%)	N/A	N/A	N/A	N/A	1 (50%)	0 (0%)	1/11 (9%)	N/A	2 (100%)
neutrophils	N/A	N/A	N/A	N/A	N/A	0 (0%)	N/A	0 (0%)	N/A	2 (100%)
histiocytes	N/A	N/A	N/A	N/A	N/A	N/A	N/A	N/A	N/A	2 (100%)
small lymphocytes (reactive)	4 (100%)	N/A	N/A	N/A	1 (100%)	2 (100%)	N/A	N/A	N/A	N/A
plasma cells	4 (100%)	N/A	N/A	N/A	N/A	N/A	N/A	N/A	N/A	N/A
**immunophenotype**										
ALK	N/A	N/A	0 (0%)	0 (0%)	N/A	0 (0%)	N/A	N/A	N/A	N/A
CD2+	4 (100%)	6/6 (100%)	1 (100%)	2 (100%)	N/A	N/A	N/A	N/A	1 (100%)	2 (100%)
CD3+	4 (100%)	8 (88%)	1 (100%)	2 (100%)	0 (0%)	2 (100%)	1 (100%)	N/A	1 (100%)	2 (100%)
CD5+	N/A	5/8 (62,5%)	1 (100%)	2 (100%)	N/A	0 (0%)	N/A	N/A	1 (100%)	N/A
**CD4+**/CD8-	0 (0%)	0 (0%)	0 (0%)	0 (0%)	0 (0%)	0 (0%)	0 (0%)	0 (0%)	0 (0%)	0 (0%)
CD4-/**CD8+**	4 (100%)	8 (88,9%)	1 (100%)	0 (0%)	1 (100%)	2 (100%)	1 (100%)	11/11 (100%)	1 (100%)	2 (100%)
**CD4+/CD8+**	0 (0%)	0 (0%)	0 (0%)	2 (100%) +/-	0 (0%)	0 (0%)	0 (0%)	0 (0%)	0 (0%)	0 (0%)
CD4-/CD8-	0 (0%)	1 (11,1%)	0 (0%)	0 (0%)	0 (0%)	0 (0%)	0 (0%)	0 (0%)	0 (0%)	0 (0%)
CD15+	N/A	N/A	N/A	N/A	N/A	N/A	N/A	N/A	N/A	N/A
CD20+	N/A	N/A	0 (0%)	N/A	0 (0%)	N/A	N/A	N/A	0 (0%)	0 (0%)
CD30+	4 (100%)	8 (88%)	1 (100%)	1 (50%)	1 (100%)	2 (100%)	1 (100%)	11/11 (100%)	1 (100%)	0 (0%)
CD45+	N/A	N/A	1 (100%) CD45R0	N/A	N/A	N/A	1 (100%) CD45R0	N/A	N/A	2 (100%) CD45R0
CD56+	0 (0%)	2 (22%)	0 (0%)	2 (100%)	0 (0%)	0 (0%)	1 (100%)	N/A	1 (100%)	0 (0%)
Ki-67	N/A	N/A	N/A	N/A	N/A	N/A	N/A	N/A	N/A	N/A
TCR-alpha/beta	N/A	8/9 (89%)	N/A	N/A	N/A	N/A	N/A	N/A	N/A	2 (100%)
TCR-gamma/delta	N/A	N/A	N/A	N/A	N/A	N/A	1 (100%)	N/A	N/A	N/A
TIA-1/ granzyme B/perforin	4 (100%)	9 (100%)	1 (100%)	2 (100%)	1 (100%)	2 (100%)	N/A	N/A	1 (100%)	2 (100%)
DUSP22-IRF4	N/A	N/A	N/A	N/A	N/A	N/A	N/A	N/A	N/A	N/A

	Mani (2016) (85)	Marshalko (2016) (23)	Matoula (2016) (70)	Barrett (2016) (69)	Hori-Kosogabe (2018) (71)	Doré Badje (2019) (72)	Simo (2019) (73)	Pavendeep (2021) (74)	Bergqvist (2021) (75)	Total
**number of examined specimen**	1	7	1	1	1	1	7	1	14	74
**number of patients**	1	7	1	1	1	1	4	1	14	71
**age of patients (years)**										
mean	27	32	38	13	40	46	57	51	48,5	41,28
min	27	5	38	13	40	46	43	51	?	5,00
max	27	58	38	13	40	46	63	51	?	91,00
**sex of patients**										
male	1	1	0	1	1	0	3	1	9	38
female	0	6	1	0	0	1	1	0	5	32
divers	0	0	0	0	0	0	0	0	0	0
**histological features**										
epidermotropic (basal)	1 (100%)	7 (100%)	1 (100%)	1 (100%)	1 (100%)	1 (100%)	6 (86%)	1 (100%)	12 (86%)	57/67 (85%)
epidermotropic (pagetoid)	1 (100%)	7 (100%)	1 (100%)	1 (100%)	1 (100%)	1 (100%)	3 (43%)	1 (100%)	2 (14%)	43/64 (67%)
dermal	1 (100%)	7 (100%)	1 (100%)	1 (100%)	1 (100%)	1 (100%)	7 (100%)	1 (100%)	14 (100)	68/68 (100%)
perifollicular	1 (100%)	1 (14%)	N/A	N/A	N/A	N/A	7 (100%)	N/A	N/A	16/28 (57%)
folliculotropic	N/A	1 (14%)	N/A	N/A	N/A	N/A	N/A	N/A	N/A	1/7 (14%)
angiocentric	1 (100%)	3 (43%)	N/A	1 (100%)?	N/A	N/A	N/A	N/A	1 (7%)	10/31 (32%)
perivascular	1 (100%)	5 (71%)	1 (100%)	1 (100%)	N/A	N/A	7 (100%)	N/A	7 (50%)	51/63 (81%)
vascular occlusion	N/A	N/A	N/A	1 (100%)?	N/A	N/A	N/A	N/A	N/A	1/3 (33%)
hemorrhage	N/A	3 (43%)	N/A	N/A	N/A	N/A	4 (57%)	N/A	N/A	16/30 (53%)
granulomatous	N/A	N/A	N/A	N/A	N/A	N/A	N/A	N/A	N/A	3/23 (13%)
perisyringo	1 (100%)	3 (43%)	N/A	N/A	N/A	N/A	7 (100%)	N/A	N/A	18/28 (64%)
syringotropic	N/A	N/A	N/A	N/A	N/A	N/A	N/A	N/A	N/A	N/A
diffuse Infiltrate	1 (100%)	1 (14%)	1 (100%)	1 (100%)	1 (100%)	1 (100%)	7 (100%)	N/A	N/A	38/44 (36%)
single Infiltrate	N/A	0 (0%)	0 (0%)	0 (0%)	N/A	N/A	0 (0%)	N/A	N/A	2/37 (5%)
cluster	N/A	1 (14%)	N/A	N/A	N/A	N/A	N/A	N/A	N/A	4/28 (14%)
nodular	N/A	N/A	N/A	N/A	N/A	N/A	N/A	N/A	N/A	4/17 (24%)
cohesive sheets	N/A	N/A	1 (100%)	N/A	N/A	N/A	N/A	N/A	0 (0%)	3/25 (8%)
small cells	N/A	4 (57%)	1 (100%)	1 (100%)	0 (0%)	0 (0%)	2 (29%)	0 (0%)	3 (21%)	26/63 (41%)
medium cells	N/A	4 (57%)	1 (100%)	1 (100%)	0 (0%)	1 (100%)	7 (100%)	0 (0%)	8 (57%)	41/66 (62%)
large cells	N/A	2 (29%)	1 (100%)	1 (100%)	1 (100%)	1 (100%)	5 (71%)	1 (100%)	2 (14%)	28/64 (44%)
monomorph cells	N/A	N/A	N/A	N/A	N/A	N/A	N/A	N/A	0 (0%)	6/43 (14%)
pleomorph cells	N/A	N/A	N/A	N/A	N/A	N/A	N/A	N/A	14 (100%)	37/44 (84%)
immunoblastic cells	N/A	N/A	N/A	N/A	N/A	N/A	N/A	N/A	N/A	0/10 (0%)
anaplastic cells	N/A	N/A	N/A	N/A	N/A	N/A	N/A	N/A	0 (0%)	1/28 (4%)
multinucleated cells (Reed-Sternberg-like)	N/A	N/A	N/A	N/A	N/A	N/A	N/A	N/A	N/A	1/15 (7%)
eosinophils	0 (0%)	1 (14%)	N/A	1 (100%)	N/A	N/A	N/A	N/A	3 (21%)	13/43 (30%)
neutrophils	0 (0%)	N/A	N/A	1 (100%)	1 (100%)	N/A	N/A	N/A	3 (21%)	7/38 (18%)
histiocytes	1 (100%)	0 (0%)	N/A	N/A	N/A	N/A	N/A	N/A	N/A	3/9 (33%)
small lymphocytes (reactive)	1 (100%)	N/A	N/A	N/A	N/A	N/A	N/A	N/A	N/A	8/8 (100%)
plasma cells	1 (100%)	0 (0%)	N/A	N/A	N/A	N/A	N/A	N/A	N/A	5/12 (42%)
**immunophenotype**										
ALK	N/A	N/A	N/A	N/A	N/A	N/A	N/A	0 (0%)	N/A	0/6 (0%)
CD2+	N/A	N/A	N/A	N/A	N/A	1 (100%)	6/6 (100%)	N/A	13 (93%)	36/37 (97%)
CD3+	1 (100%)	N/A	1 (100%)	N/A	1 (100%)	1 (100%)	6 (86%)	1 (100%)	13 (93%)	45/49 (92%)
CD5+	0 (0%)	N/A	N/A	N/A	N/A	1 (100%)	0 (0%)	0 (0%)	8 (57%)	18/38 (47%)
**CD4+**/CD8-	0 (0%)	0 (0%)	0 (0%)	0 (0%)	0 (0%)	0 (0%)	0 (0%)	0 (0%)	0 (0%)	0/68 (0%)
CD4-/**CD8+**	1 (100%)	7 (100%)	1 (100%)	1 (100%)	1 (100%)	1 (100%)	7 (100%)	1 (100%)	13 (93%)	64/68 (94%)
**CD4+/CD8+**	0 (0%)	0 (0%)	0 (0%)	0 (0%)	0 (0%)	0 (0%)	0 (0%)	0 (0%)	1 (7%)	3/68 (4%)
CD4-/CD8-	0 (0%)	0 (0%)	0 (0%)	0 (0%)	0 (0%)	0 (0%)	0 (0%)	0 (0%)	0 (0%)	1/68 (2%)
CD15+	N/A	N/A	N/A	N/A	N/A	N/A	N/A	N/A	N/A	N/A
CD20+	0 (0%)	N/A	N/A	N/A	N/A	N/A	N/A	N/A	N/A	0/6 (0%)
CD30+	1 (100%)	7 (100%)	1 (100%)	1 (100%)	1 (100%)	1 (100%)	5 (71%)	1 (100%)	14 (100%)	62/68 (91%)
CD45+	N/A	N/A	N/A	N/A	N/A	1 (100%)	N/A	N/A	N/A	4/4 (100%)
CD56+	1 (100%)	N/A	N/A	N/A	N/A	1 (100%)	0 (0%)	0 (0%)	5 (36%)	13/47 (28%)
Ki-67	N/A	N/A	N/A	N/A	N/A	N/A	N/A	N/A	N/A	N/A
TCR-alpha/beta	N/A	N/A	N/A	N/A	N/A	0 (0%)	N/A	N/A	N/A	10/12 (83%)
TCR-gamma/delta	N/A	N/A	N/A	N/A	N/A	1 (100%)	N/A	N/A	0/2 (0%)	2/4 (50%)
TIA-1/ granzyme B/perforin	1 (100%)	N/A	N/A	N/A	1 (100%)	1 (100%)	5/7 (71%)	N/A	7 (50%)	37/46 (80%)
DUSP22-IRF4	N/A	N/A	N/A	N/A	N/A	N/A		N/A	0/9 (0%)	0/9 (0%)

Suppl. Table S5: All investigated histologic patterns, immunophenotypic and genetic features, which could be extracted from 12 articles with diagnosed Lyp subtype E cases. The highlighted lines show the key-features we extracted showed in the figures.

	Wu (2004) (76)	Kempf (2013) (9)	Sharaf (2014) (28)	Kiavash (2015) (29)	Uchiyama (2016) (77)	Pindado-Ortega (2017) (80)	Belousova (2018) (78)	Fujimura (2016) (79)	Lee (2019) (82)	Ba (2019) (81)	Rajashekara Swamy (2021) (83)	Bergqvist (2021) (75)	Total
**number of examined specimen**	1	21	1	1	1	1	1	1	1	1	1	10	41
**number of patients**	1	16	1	1	1	1	1	1	1	1	1	10	36
**age of patients (y)**													
mean	40	53,7	73	72	28	69	34	76	58	36	21	43	50,31
min	40	8	73	72	28	69	34	76	58	36	21	?	8
max	40	77	73	72	28	69	34	76	58	36	21	?	77
**sex of patients**													
male	0	12	0	1	1	1	1	0	1	0	1	6	24
female	1	4	1	0	0	0	0	1	0	1	0	4	12
divers	0	0	0	0	0	0	0	0	0	0	0	0	0
**histological features**													
epidermotropic (basal)	N/A	9 (43%)	N/A	1 (100%)	0 (0%)	0 (0%)	0 (0%)	0 (0%)	1 (100%)	0 (0%)	0 (0%)	0 (0%)	11/39 (28%)
epidermotropic (pagetoid)	N/A	0 (0%)	N/A	0 (0%)	0 (0%)	0(0%)	0 (0%)	0 (0%)	1 (100%)	0 (0%)	0 (0%)	0 (0%)	1/39 (3%)
dermal	1 (100%)	21 (100%)	1 (100%)	1 (100%)	1 (100%)	1 (100%)	1 (100%)	1 (100%)	1 (100%)	1 (100%)	1 (100%)	10 (100%)	41/41 (100%)
perifollicular	N/A	N/A	N/A	N/A	N/A	N/A	N/A	N/A	N/A	N/A	N/A	N/A	N/A
folliculotropic	N/A	3 (14%)	N/A	N/A	N/A	N/A	N/A	N/A	N/A	N/A	N/A	N/A	3/21 (14%)
angiocentric	1 (100%)	21 (100%)	1 (100%)	1 (100%)	1 (100%)	1 (100%)	1 (100%)	1 (100%)	1 (100%)	1 (100%)	1 (100%)	3 (30%)	34/41 (83%)
perivascular	1 (100%)	21 (100%)	1 (100%)	1 (100%)	1 (100%)	1 (100%)	1 (100%)	1 (100%)	1 (100%)	1 (100%)	1 (100%)	7 (70%)	38/41 (93%)
vascular occlusion	1 (100%)	21 (100%)	1 (100%)	1 (100%)	1 (100%)	1 (100%)	1 (100%)	N/A	1 (100%)	1 (100%)	1 (100%)	N/A	30/30 (100%)
hemorrhage	1 (100%)	16 (76%)	1 (100%)	1 (100%)	N/A	1 (100%)	1 (100%)	1 (100%)	1 (100%)	1 (100%)	1 (100%)	N/A	25/30 (83%)
granulomatous	N/A	N/A	N/A	N/A	N/A	N/A	N/A	N/A	N/A	N/A	N/A	N/A	N/A
perisyringo	N/A	N/A	N/A	N/A	N/A	N/A	N/A	N/A	N/A	N/A	N/A	N/A	N/A
syringotropic	N/A	8 (38%)	N/A	N/A	N/A	N/A	N/A	N/A	N/A	N/A	N/A	N/A	8/21 (38%)
diffuse Infiltrate	N/A	N/A	N/A	1 (100%)	N/A	N/A	N/A	N/A	N/A	N/A	N/A	N/A	1/1 (100%)
single Infiltrate	N/A	N/A	N/A	N/A	N/A	1 (100%)	N/A	N/A	N/A	N/A	N/A	N/A	1/1 (100%)
cluster	N/A	N/A	N/A	N/A	N/A	N/A	N/A	N/A	N/A	N/A	N/A	N/A	N/A
nodular	N/A	N/A	N/A	N/A	N/A	N/A	1 (100%)	N/A	N/A	N/A	1 (100%)	N/A	2/2 (100%)
cohesive sheets	N/A	N/A	N/A	N/A	N/A	N/A	N/A	N/A	N/A	N/A	N/A	1 (10%)	1/10 (3%)
small cells	0 (0%)	21 (100%)	0 (0%)	N/A	1 (100%)	1 (100%)	0 (0%)	N/A	1 (100%)	0 (0%)	1 (100%)	0 (0%)	25/39 (64%)
medium cells	1 (100%)	21 (100%)	1 (100%)	N/A	1 (100%)	1 (100%)	1 (100%)	N/A	1 (100%)	1 (100%)	1 (100%)	8 (80%)	37/39 (95%)
large cells	0 (0%)	21 (100%)	1 (100%)	1 (100%)	1 (100%)	0 (0%)	1 (100%)	N/A	1 (100%)	1 (100%)	1 (100%)	2 (20%)	30/40 (75%)
monomorph cells	N/A	N/A	N/A	N/A	N/A	N/A	0 (0%)	N/A	N/A	N/A	N/A	1 (10%)	1/11 (9%)
pleomorph cells	N/A	21 (100%)	1 (100%)	N/A	N/A	N/A	1 (100%)	N/A	N/A	N/A	N/A	9 (90%)	32/33 (97%)
immunoblastic cells	N/A	N/A	N/A	N/A	N/A	N/A	N/A	N/A	N/A	N/A	N/A	N/A	N/A
anaplastic cells	N/A	N/A	N/A	N/A	N/A	N/A	N/A	N/A	N/A	N/A	N/A	1 (10%)	1/10 (10%)
multinucleated cells (Reed-Sternberg-like)	N/A	N/A	N/A	N/A	N/A	N/A	N/A	N/A	N/A	N/A	N/A	N/A	N/A
eosinophils	N/A	16 (76%)	0 (0%)	1 (100%)	N/A	1 (100%)	0 (0%)	N/A	N/A	N/A	N/A	4 (40%)	22/35 (63%)
neutrophils	1 (100%)	21 (100%)	1 (100%)	1 (100%)	N/A	1 (100%)	1 (100%)	N/A	N/A	N/A	N/A	4 (40%)	30/36 (83%)
histiocytes	N/A	N/A	1 (100%)	N/A	N/A	0 (0%)	1 (100%)	N/A	N/A	N/A	N/A	N/A	2/3 (67%)
small lymphocytes (reactive)	1 (100%)	N/A	1 (100%)	N/A	N/A	1 (100%)	1 (100%)	N/A	N/A	1 (100%)	1 (100%)	10 (100%)	16/16 (100%)
plasma cells	N/A	N/A	N/A		N/A	0 (0%)	0 (0%)	N/A	N/A	N/A	N/A	N/A	0/2 (0%)
**immunophenotype**													
ALK+	N/A	0/16 (0%)	N/A	0 (0%)	0 (0%)	N/A	0 (0%)	N/A	0 (0%)	0 (0%)	N/A	N/A	0/21 (0%)
CD2+	N/A	14/14 (100%)	N/A	1 (100%)	N/A	N/A	N/A	N/A	N/A	N/A	N/A	10 (100%)	25/25 (100%)
CD3+	1 (100%)	13/14 (93%)	N/A	1 (100%)	N/A	1 (100%)	1 (100%)	1 (100%)	N/A	1 (100%)	1 (100%)	7 (70%)	27/31 (87%)
CD5+	0 (0%)	13/16 (81%)	N/A	1 (100%)	N/A	0 (0%)	1 (100%)	N/A	N/A	0 (0%)	N/A	7 (70%)	22/31 (71%)
**CD4+**/CD8-	0 (0%)	4/16 (25%)	0 (0%)	0 (0%)	0 (0%)	0 (0%)	1 (100%)	0 (0%)	1 (100%)	0 (0%)	0 (0%)	3 (30%)	9/36 (25%)
CD4-/**CD8+**	1 (100%)	8/16 (50%)	0 (0%)	0 (0%)	1 (100%)	1 (100%)	0 (0%)	1 (100%)	0 (0%)	0 (0%)	0 (0%)	4 (40%)	16/36 (44%)
**CD4+/CD8+**	0 (0%)	4/16 (25%)	1 (100%)	1 (100%)	0 (0%)	0 (0%)	0 (0%)	0 (0%)	0 (0%)	0 (0%)	1 (100%)	3 (30%)	10/36 (28%)
CD4-/CD8-	0 (0%)	0/16 (0%)	0 (0%)	0 (0%)	0 (0%)	0 (0%)	0 (0%)	0 (0%)	0 (0%)	1 (100%)	0 (0%)	0 (0%)	1/36 (3%)
CD15+	N/A	N/A	N/A	N/A	N/A	N/A	N/A	N/A	N/A	N/A	N/A	N/A	N/A
CD20+	0 (0%)	N/A	0 (0%)	N/A	N/A	N/A	1 (100%) (CD20+ B-cells	N/A	N/A	0 (0%)	0 (0%)	N/A	1/5 (20%)
CD30+	1 (100%)	15/15 (100%)	1 (100%)	1 (100%)	1 (100%)	1 (100%)	1 (100%)	1 (100%)	1 (100%)	1 (100%)	1 (100%)	10 (100%)	36/36 (100%)
CD45+	N/A	6/9 (67%) CD45R0	N/A	N/A	N/A	N/A	N/A	N/A	N/A	N/A	N/A	N/A	6/9 (67%)
CD56+	1 (100%)	3/13 (23%)	0 (0%)	0 (0%)	0 (0%)	0 (0%)	0 (0%)	0 (0%)	N/A	1 (100%)	0 (0%)	2/7 (28,6%)	7/29 (24%)
Ki-67	N/A	N/A	N/A	N/A	N/A	N/A	N/A	N/A	N/A	N/A	N/A	N/A	N/A
TCR-alpha/beta	N/A	13/13 (100%)	N/A	N/A	N/A	N/A	N/A	N/A	1/1 (100%)	N/A	N/A	N/A	14/14 (100%)
TCR-gamma/delta	N/A	0/10 (0%)	N/A	N/A	N/A	N/A	N/A	N/A	N/A	N/A	N/A	0/2 (0%)	0/12 (0%)
TIA-1/granzyme B/perforin	1 (100%)	9/11 (82%)	1 (100%)	0 (0%)	1 (100%)	0 (0%)	1 (100%)	N/A	N/A	0 (0%)	N/A	6 (60%)	19/24 (79%)
DUSP22-IRF4	N/A	N/A	N/A	N/A	N/A	N/A	N/A	N/A	N/A	N/A	N/A	0/4 (0%)	0/4 (0%)

Suppl. Table S6: All investigated histologic patterns, immunophenotypic and genetic features, which could be extracted from 2 articles with diagnosed Lyp subtype with 6p25.3. The highlighted lines show the key-features we extracted showed in the figures.

	Karai (2013) (30)	Kluk (2014) (84)	Total
**number of examined specimen**	11	1	12
**number of patients**	11	1	12
**age of patients (y)**			
mean	75	67	71
min	67	67	67
max	88	67	88
**sex of patients**			
male	9	0	9
female	2	1	3
divers	0	0	0
**histological features**			
epidermotropic (basal)	11 (100%)	1 (100%)	12/12 (100%)
epidermotropic (pagetoid)	11 (100%)	1 (100%)	12/12 (100%)
dermal	11 (100%)	1 (100%)	12/12 (100%)
perifollicular	9 (82%)	N/A	9/11 (82%)
folliculotropic	N/A	N/A	N/A
angiocentric	N/A	N/A	N/A
perivascular	N/A	N/A	N/A
vascular occlusion	N/A	N/A	N/A
hemorrhage	N/A	N/A	N/A
granulomatous	N/A	N/A	N/A
perisyringo	9 (82%)	N/A	9/11 (82%)
syringotropic	N/A	N/A	N/A
diffuse Infiltrate	11 (100%)	0 (0%)	11/12 (92%)
single Infiltrate	N/A	0 (0%)	0/1 (0%)
cluster	N/A	0 (0%)	0/1 (0%)
nodular	N/A	1 (100%)	1/1 (100%)
cohesive sheets	N/A	N/A	0/0 (0%)
small cells	11 (100%)	1 (100%)	12/12 (100%)
medium cells	11 (100%)	1 (100%)	12/12 (100%)
large cells	11 (100%)	1 (100%)	12/12 (100%)
monomorph cells	N/A	0 (0%)	0/1 (0%)
pleomorph cells	N/A	0 (0%)	0/1 (0%)
immunoblastic cells	N/A	N/A	N/A
anaplastic cells	N/A	1 (100%)	1/1 (100%)
multinucleated cells (Reed-Sternberg-like)	11 (100%)	1 (100%)	12/12 (100%)
eosinophils	2 (18%)	N/A	2/11 (18%)
neutrophils	N/A	N/A	N/A
histiocytes	N/A	N/A	N/A
small lymphocytes (reactive)	N/A	N/A	N/A
plasma cells	N/A	N/A	N/A
**immunophenotype**			
ALK	0 (0%)	0 (0%)	0/12 (0%)
CD2+	N/A	0 (0%)	0/1 (0%)
CD3+	10 (90%)	1 (100%)	11/12 (92%)
CD5+	N/A	1 (100%)	1/1 (100%)
**CD4+**/CD8-	1 (9,1%)	0 (0%)	1/12 (8%)
CD4-/**CD8+**	4 (36,3%)	0 (0%)	4/12 (33%)
**CD4+**/**CD8+**	1 (9,1%)	0 (0%)	1/12 (8%)
CD4-/CD8-	5 (45,5%)	1 (100%)	6/12 (50%)
CD15+	6 (54%)	N/A	6/11 (54%)
CD20+	N/A	N/A	N/A
CD30+	11 (100%)	1 (100%)	12/12 (100%)
CD45+	N/A	N/A	N/A
CD56+	N/A	0 (0%)	0/1 (0%)
Ki-67	60-80%, >80%	>95%	-
TCR-alpha/beta	N/A	N/A	N/A
TCR-gamma/delta	N/A	N/A	N/A
TIA-1/granzyme B/perforin	2 (18%)	0 (0%)	2/12 (17%)
DUSP22-IRF4	11 (100%)	1 (100%)	12/12 (100%)
